# A pathway for protective quenching in antenna proteins of Photosystem II

**DOI:** 10.1038/s41598-017-02892-w

**Published:** 2017-05-31

**Authors:** Sotiris Papadatos, Antreas C. Charalambous, Vangelis Daskalakis

**Affiliations:** Department of Environmental Science and Technology, Cyprus University of Technology, 30 Archbishop Kyprianou Str., 3603 Limassol, Cyprus

## Abstract

Photosynthesis is common in nature, converting sunlight energy into proton motive force and reducing power. The increased spectral range absorption of light exerted by pigments (i.e. chlorophylls, Chls) within Light Harvesting Complexes (LHCs) proves an important advantage under low light conditions. However, in the exposure to excess light, oxidative damages and ultimately cell death can occur. A down-regulatory mechanism, thus, has been evolved (non-photochemical quenching, NPQ). The mechanistic details of its major component (qE) are missing at the atomic scale. The research herein, initiates on solid evidence from the current NPQ *state of the art*, and reveals a detailed atomistic view by large scale Molecular Dynamics, Metadynamics and *ab initio* Simulations. The results demonstrate a complete picture of an elaborate common molecular design. All probed antenna proteins (major LHCII from spinach-pea, CP29 from spinach) show striking plasticity in helix-D, under NPQ conditions. This induces changes in Qy bands in excitation and absorption spectra of the near-by pigment pair (Chl613-614) that could emerge as a new quenching site. Zeaxanthin enhances this plasticity (and possibly the quenching) even at milder NPQ conditions.

## Introduction

**Figure 1:**
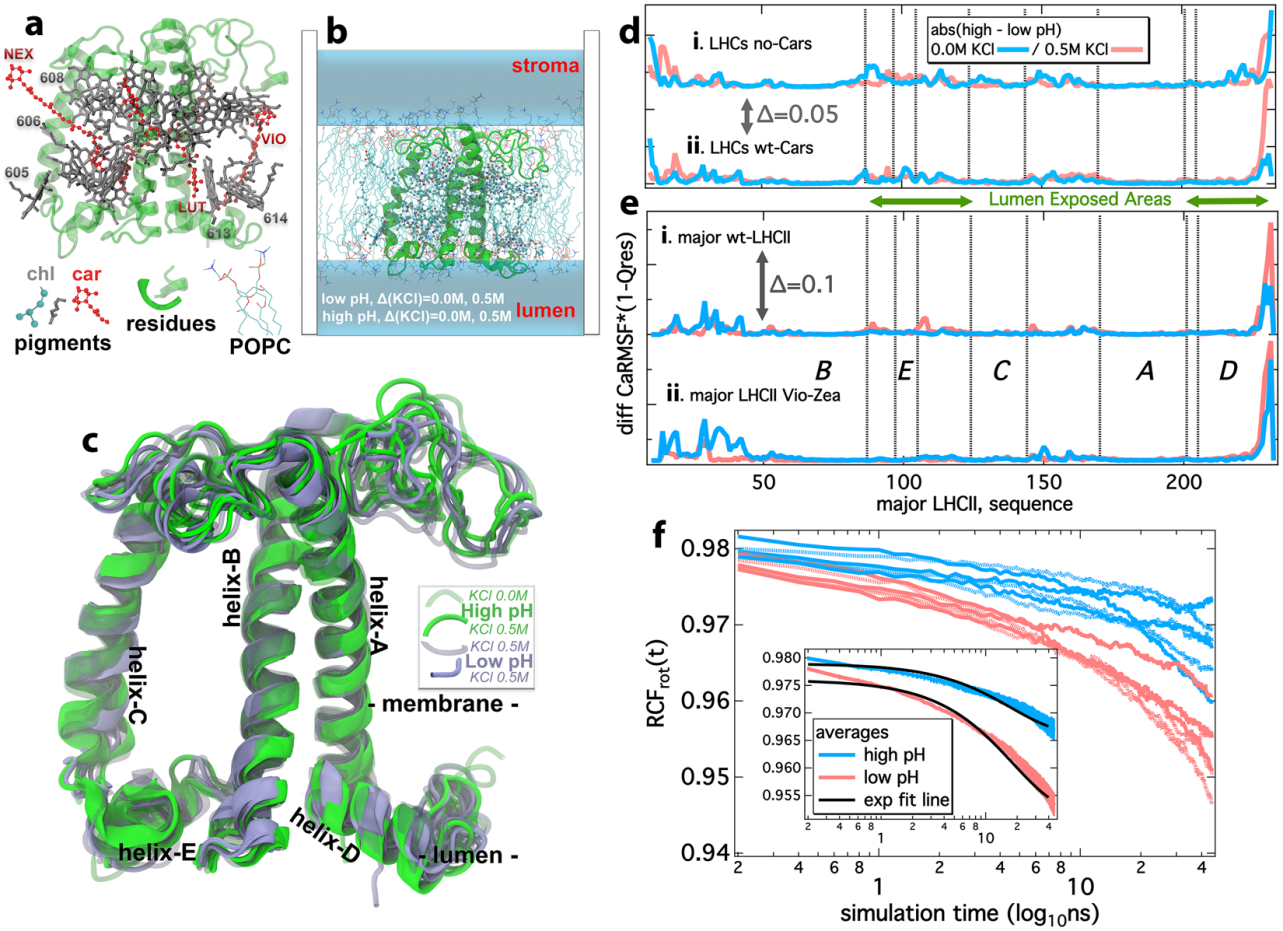
The Light Harvesting Complex structure and dynamics in response to membrane energization by proton and ionic gradients. (**a**) The crystal structure of the major LHCII from spinach (pdb code: 1RWT). The residues are shown in green, the pigments in gray (Chls – Chlorophylls) and red (Cars – Carotenoids). Selected Chl groups are numbered based on the crystal structure and carotenoids are labeled: Neoxanthin (Nex), Violaxanthin (Vio) and Lutein (Lut)^2^. (**b**) The major LHCII embedded in a lipid bilayer membrane with varying lumen ionic strengths. (**c**) The average structures out of the clustering analysis for all wild-type (wt – with Vio) samples from the CMD trajectories (pigments have been omitted for clarity). A preference for the conformation towards the membrane for helix-D is evident, upon the membrane maximum energization (solid ice blue conformations). The different helices are designated in black. (**d**,**e**) The product of Ca-RMSF * (1-Qres) in absolute high minus low pH difference in the presence (red) or in the absence (blue) of a Δ(KCl). The peaks indicate regions of high flexibility and distinct structural conformation for the different samples. Traces **i**–**ii** in panel d refer to averages over all LHCs (major from spinach-pea and the minor CP29), without (no-Cars) and with (wt-Cars) carotenoids. Traces **i–ii** in panel e refer only to the major LHCII (spinach, pea) in the wt-forms and the Violaxanthin (Vio)−Zeaxanthin (Zea) replacement form. The different helices are designated in black italics within the graph. (**f**) The rotational correlation functions (RCF_rot_) of the backbone atoms in the loop connecting helices A and D (residues 199–207 for the major LHCII from spinach and pea, and 216–224 for CP29 from spinach) upon the transition from lower (blue) to higher (red) lumen ΔpH in the presence and absence of Δ(KCl). In the inset we depict the average RCF_rot_ with the line widths to represent the standard deviation out of the averaging. The black lines in the inset correspond to the exponential decay fitting.

Photosystem II (PSII) is one of the most important biochemical nanomachines in nature and part of the photosynthetic apparatus of higher plants. It consists of the reaction center (RC), and the light harvesting complexes (LHCs): the proximal (CP43, CP47), and the outer (major LHCII, CP24, CP26 and CP29) antenna^1^. The crystal structure of the major LHCII from spinach^2^ is shown in Fig. 1a and consists mainly of a protein matrix (green cartoons) and 18 pigments (14 Chlorophylls-Chls in gray and 4 Carotenoid molecules-Cars in red), embedded in a lipid bilayer membrane (Fig. 1b). The pigments harvest the photon energy in an increased spectral range and get promoted to an electronically excited state^3^. The non-radiative relaxation of high-energy excited states of the Chls in LHCs to the lowest excited states triggers Photosynthesis. The exciton transfer from the antenna pigments to the RC of the photosystems powers the water oxidation that leads to O_2_ evolution. In addition, water splitting drives the electron transfer from H_2_O to NADPH and the vectorial proton release that contributes to the *proton motive force* (*pmf*) in the thylakoid membranes of the chloroplasts required for the ATP synthesis.

The increased spectral range absorption of light by the LHCs proves an important advantage under low light conditions, but in the exposure to excess light, oxidative damages and ultimately cell death can occur. This impairs the native biochemical components of the apparatus and compromises the efficient energy storage. Despite the deep understanding of the photochemical catalytic cycle in PSII we have a rather inadequate understanding of its down-regulation process that protects the apparatus (photoprotection). The down-regulation mechanism in PSII, called Non-Photochemical Quenching (NPQ), has an energy dependent major component (qE) that is co-regulated by low lumen pH (ΔpH)^4^ and ion fluxes (K^+^, Ca^2+^, Mg^2+^, Cl^−^) (ΔΨ) in Lumen-Stroma areas, but in diverse ways with the direct ion effects still under debate^5^. In addition, Violaxanthin De-epoxidase (VDE) is activated by the acidification of the lumen space. VDE catalyzes the Violaxanthin (Vio) to Zeaxanthin (Zea) conversion (the xanthophyll cycle) and changes the carotenoid content of the LHCs, or thylakoid membranes under NPQ conditions^6, 7^. Zea controls the qE quenching amplitude, its acceleration-relaxation, as well as the shift of qE triggering to higher lumen pH values, but the atomic-scale details remain obscure^8–10^. In addition, Zea increases the pigment-pigment coupling in conditions mimicking high light intensity^11^.

ΔpH, ΔΨ and Zea have been associated with conformational changes in the LHCII. Even small conformational changes within LHCII can affect significantly the excited energies and quenching dynamics of the pigments^12^. However, both the specific conformational changes within the LHCs and the actual quenching site(s) remain elusive^13–15^, with the underlying picture at the molecular level to not have been resolved yet^16, 17^. Indeed, the pursue of such a molecular mechanism proves an important aspect that made PSII the focus of many research groups and a matter of considerable debate in the ongoing challenge to fundamental scientific research^13^. It is well established that the energies and quenching dynamics of the pigment excited states within LHCII are regulated by pigment-protein interactions. The quantum coherent and incoherent energy transfer plays a major role in the process^18, 19^. In addition, the highly debated quenching site involves pigment-pigment interactions. For example, (i) the incoherent exciton transfer from the Chl to the short lived S_1_ state of Cars has been implicated in the NPQ response^20^, i.e between Lutein 1 (Lut1) and the adjacent Chl-a, that interact more closely in an energy dissipating conformation^21^, (ii) Chl-Chl quenching states based on a charge-transfer character^22–24^ have also been implicated in quenching, (iii) the neoxanthin (Neo) and the adjacent Chl-b, that interact more closely in an energy dissipating form^25, 26^, (iv) the Chl-614 pigment near the helix-D of the LHCs (Fig. 1a), where a conformational change in the helix has been proposed to alter the adjacent Chl macrocycle to a dissipating form^27, 28^. But what could be the cascade of events that leads from the well-established lumen acidification that triggers qE, to the obscure protein conformational changes and finally to the quenching site(s) and dynamics ? Our working hypothesis is that under different lumen perturbations (ΔpH and ΔΨ gradients), the active player (LHCs) domains related to NPQ will exert high flexibility, or will assume distinct structures upon the binding of Zea, where the pigment-pigment, or protein-pigment interactions are altered. At this stage, important questions emerge: (**i**) what is the response of different LHCs to the lumen acidification (ΔpH), ion fluxes (ΔΨ) and Zea binding? What are the specific domains that respond and to what extent ? (**ii**) How are these domains affecting the adjacent pigment sites? (**iii**) What is the role of the pigment packing, within the protein matrices of the LHCs, on the quenching ? (**iv**) What is the related mechanism (cascade of events) from the ΔpH − ΔΨ induction to quenching that involves these sites ? To answer these questions, it is important to have an atomic-scale resolution of the LHCs − ΔpH/ΔΨ − Zea relations by Molecular Simulations. The knowledge of a structure and dynamics, at the atomic level, can be used to reconstruct experimental observables in a complex connection between structure, experimental measurements and function of LHCII under NPQ conditions.

Large scale all-atom Classical Molecular Dynamics (CMD), Metadynamics and full Quantum (*ab initio*) simulations are employed herein, as powerful tools^16, 28–32^, to give insight into the LHCs response under ΔpH − ΔΨ^5^ and the Vio−Zea conversion^6^, in terms of induced conformational changes that can then be associated with affected quenching sites. We simulate the NPQ conditions by a perturbation in the thylakoid lumen, adding H^+^, and salt (KCl) gradients^28^. Four Lumen states are probed per LHC: low pH < 6 (higher energization) and high pH > 6 (lower energization), with a ±1 tolerance in combination with the Δ(KCl) gradient (lumen – stroma) at 0.0 and 0.5 M^28^. Τhree different membrane-embedded LHCs models are built in such way (major LHCII from spinach and pea and minor CP29 from spinach) of around 40,000 atoms each, including the LHCII complex, a membrane patch of between 120–128 united-atom UA-POPC lipids and 8500 TIP3P^33^ water molecules at 6.5 × 6.5 nm^2^ lateral dimensions. Separate models with a Vio−Zea conversion, additionally introduced into the major LHCII from spinach at low pH, mimic stress response^34^ and the VDE activity under NPQ^6, 7^. Car-deprived models were also probed only for comparison. In any case, when results related to the latter models are presented, we explicitly mention that these are based on Car-deprived structures. However, the main results of this study are based on Car-rich structures. The cumulative production simulation time was at 15 μs. For further details refer to the Materials and Methods Section and the Supplementary Information (SI).

A striking plasticity is identified in the helix-D region of the probed LHCs, under NPQ conditions. This is associated with changes (redshifts) in the Qy bands in excitation and absorption spectra of the near-by pair of pigments (Chl613-614) at the Time Dependent Density Functional Theory (TDDFT) level^35^. This pair emerges as a potential new quenching site. Zeaxanthin presence enhances this plasticity, and focuses the response at the helix-D region, even at lower thylakoid membrane energizations (milder NPQ conditions).

## Results

### LHC conformations under NPQ conditions

The presence of Cars affects the protein response, under NPQ conditions (high membrane energization), especially under a Δ(KCl) at 0.5 M, by enabling lower alpha carbon Root Mean Square Deviation (RMSD) values (*see* Table 3S in SI). Spinach antennas (major and minor) show relatively low maximum RMSD values in the presence of carotenoids (0.14–0.29). Especially for CP29, the maximum RMSD values are the lowest recorded. Cars presence structurally stabilizes the proteins with up to around 30% decrease in the RMSD values, compared to their absence, under high or Low lumen pH for all antenna proteins probed. Noteworthy, is thus the fact that pigment-deprived antenna proteins are relatively rigid proteins (~0.29–0.33 RMSD), but Cars presence improves this rigidity further by around 30%. This might be an evolutionary benefit, given that these proteins have to operate under extreme conditions of *pmf* and excitation pressure. Despite the fact that carotenoid-deprived structures are not representative of the LHCII system, we treat the systems also in their absence in order to reveal their function. This is a similar approach as in experimental, or theoretical mutations in protein systems, in order to reveal the role of specific residues and possibly provide insight into the evolution of the protein – enzyme.

The average structures out of the CMD trajectories (simulations with Cars) are shown in a superposition scheme in Fig. 1c. The green structures indicate conformations with high pH at the lumen side (lower energizations), whereas the ice blue structures refer to conformations with low pH at the lumen side (higher energizations). Two different conformations of helix-D are shown in Fig. 1c: one towards the membrane (M, higher energization) and one towards the lumen side water pool (L, lower energization). For the rest of the text we shall refer to the interchange between the latter states, as L-M motion, identified previously in CP29 only and also proposed in recent studies^27, 28^. The % contribution of this latter L-M motion to the overall response of LHCs in ΔpH/Δ(KCl), based on the CMD production trajectories, for the wt (Vio) and the Cars-deprived models (Table 3S in SI) indicates that the presence of Cars reduces the L-M motion at the higher membrane energization states, indicating a stabilizer effect, or a role as folding guide preventing uncontrollable protein conformational changes at the lumen side, when membrane energization increases. This is consistent with the proposed role of Cars in the literature^36^, and evaluates positively the empirical interaction potentials employed in this study.

Computational structural probes, such as the weighted average alpha-carbon (Ca) Root Mean Square Fluctuations (Ca-RMSF) and the (1-Qres) values refer to the per residue fluctuations, and the similarity of the environment per residue, respectively^37, 38^. We calculate a combined RMSF * (1-Qres) product per residue, at absolute low minus high lumen pH LHC state, in the presence or in the absence of a KCl gradient, Δ(KCl). High values of this product indicate high protein flexibility and distinct structure upon moving between samples with different lumen ionic strengths, as shown in Fig. 1d,e. The reasoning behind the use of the RMSF * (1-Qres) product is that in search for potential NPQ-related regions in the antenna proteins, such regions would respond with distinct conformation upon low minus high pH dynamics (indicating regions that respond to protonation, i.e. NPQ conditions). Sequence alignment was used to superimpose the results for all LHCs (Fig. 1d,e)^37, 39^. We also note that these results are free of thermal fluctuations as they are based on PCA analysis^40^ (for details refer to the Supplementary Information, SI). The results shown in Fig. 1d,e are consistent with a flexible protein N- terminus^41^. Our approach accurately captures and quantifies this flexibility. Striking are the results also for the helix-D and the C-terminus of the proteins in general with a strong peak in the RMSF * (1-Qres) product at the respective helix-D sequence. Without Cars, the flexibility is high for both low and high energization levels (Fig. 1d, *trace i*). In line with this view, upon removal of Cars, helix-D shows quite high values of RMSF * (1-Qres) both at high and low lumen pH. In the presence of wt-Cars (Vio), helix-D shows low flexibility at low levels of lumen energization and high flexibility at high energization (Fig. 1d, *trace ii*), functioning in line with the current NPQ working model on conformational changes in LHCs^13^. It is noteworthy that all three LHCs exert similar behavior upon ΔpH/Δ(KCl) induction, shown as averaged RMSF * (1-Qres) profiles in Fig. 1d (*trace ii*). However, we have to note that if CP29 is included in the average response (Fig. 1d, *trace ii*) more fluctuations are recorded (aminoacid sequence 80–170), compared to the major LHCII-only average response (Fig. 1e, *trace i*). This might explain a more important role of CP29 containing regions that seem to respond to chemiosmotic factors^28^.

Due to the involvement of the Vio de-epoxidation in NPQ^13^, as previously noted, we have run CMD trajectories for the major LHCII from spinach, where the Vio carotenoid has been replaced by Zea in the low lumen pH samples only (0.0 M or 0.5 M Δ(KCl)). A comparison between the dynamics of the major LHCII from spinach in the presence of Vio (major LHCII, wt) or Zea (major LHCII, Vio−Zea), is shown in Fig. 1e (*traces i*, *ii*). We observe that the Zea presence renders the response of the protein to lumen energization more focused on the helix-D region (compare the transition from *trace* **i** to **ii** in Fig. 1e, and also the results from larger-membrane systems probed below, for the same response at the helix-D region). This latter means that Zea presence has a significant effect on the helix-D region plasticity in LHCII, even under lower membrane energization (ΔpH only). As the response of helix-D has been proposed to relate with NPQ^27, 28^, this comes in agreement (i) with experimental observations that Zea shifts the relationship between NPQ (qE) and ΔpH so that quenching is activated at lower membrane energization levels^13, 42^, or (ii) with proposals that Zea binding leads to protein conformational changes^36^. The reason behind this enhanced response in the presence of Zea can be the fact that protonated carboxyl groups (at low lumen pH) of helix D lose their charge and can be accommodated closer to the thylakoid membrane, and away from the lumen side. This is especially enhanced in the case of a positively charged lumen (H^+^, K^+^), or a more hydrophobic environment in the region (Zea). Zea is the most hydrophobic carotenoid, followed by lutein and Vio. We propose that Zea presence enables and stabilizes a higher energization conformation of LHCII (providing a plausible molecular explanation on the role of Zea in NPQ). In other words, Zea enables and stabilizes the higher energization conformation of helix-D domain under milder NPQ conditions. Whether this higher energization conformation is a quenching conformation will be analyzed in the following sections. Nevertheless, this mode of Zea action is probably related also to its increased hydrophobicity, as previously reported.

We observe that the L-M motion is also a trait of all proteins studied herein at the lumen exposed region after residue 200 (major LHCII residue numbering). This common feature is exerted as the highest peak in the RMSF * (1-Qres) product at the helix-D region for different ΔpH and Δ(KCl) gradients.

### LHC response upon lumen perturbation

The dynamics described previously defined a common behavior of all LHCs probed in *equilibrium*, under variant lumen configurations and pigment compositions. To quantify their time-dependent response upon lumen energization (i.e. by H^+^ and KCl), we perturb the lumen side of the High pH/0.0 M KCl equilibrated sample *on the fly* and monitor the *out-of-equilibrium* events. This sample is considered as the lowest energization level model (Ref)^28^ and by such way we probe the wt protein (with Vio) while it transitions from a relaxed state, towards another state of equilibrium (NPQ conditions) for all wt-LHCs (with Vio). The Mean Square Displacement (MSD) profiles between the center of masses of helices in adjacent pairs are calculated upon the transition. We probe the out-of-equilibrium response, so a very short equilibration (1 ns) of the systems prior to production trajectories is performed to avoid missing important dynamics (*for details see the* SI). The A-D pair of helices exerts clearly distinct MSD/time slopes (Fig. 2S in SI), responding to both ΔpH/Δ(KCl), while for the rest of the pairs of helices, we measure a mixed response or insensitivity to the pH change. This makes helix-D the principal ΔpH/Δ(KCl) sensitive domain in LHCII, in line with a recent study on CP29^28^. In addition, helix-E, as a potential secondary sensitive site, responds only to ΔpH, in line with evidence from experiments on helix-E mutants where the helix-E structure and pigment binding are strongly compromised^43, 44^.

To quantify the previous behavior for the related motion between helices, we have calculated also the diffusion coefficients D_C_ between the center of masses of adjacent helices in pairs for all LHCs (with wt Cars) upon the perturbation at the lumen side. The averaged results are shown in 10^2^ nm^2^s^−1^ in Table 1. We note that the largest D_C_ coefficient is calculated for the A-D pair at ΔpH + Δ(KCl), which can be associated with a significant motion of helix-D only, as the corresponding A-B MSD/Dc values are relatively low. The D_C_ values for the helix-E motion are a priori increased even at the lowest energization sample (Ref – High lumen pH/0.0 M KCl), reflected also as high D_C_ values at ΔpH only in the averages for all LHCs. This also justifies helix-E potential role as a secondary sensitive site that responds to ΔpH only, as the respective Δ(KCl) Dc values appear relatively lower.

**Table 1:** The average diffusion coefficients D_C_ in 10^2^ nm^2^s^−1^ between the center of masses of the LHC helices in pairs (major from spinach-pea and minor CP29 from spinach).

Pairs of Helices	A–D	A–B	B–C	B–E	C–E
Lumen perturbation	Diffusion Coefficients Dc in 10^2^ nm^2^s^−1^				
Ref (High pH/0.0 M KCl)	83	16	32	148	100
Δ (KCl)	109 (31%)	23 (44%)	15 (−53%)	93 (−37%)	114 (14%)
ΔpH	161 (94%)	49 (206%)	41 (28%)	204 (38%)	194 (94%)
ΔpH + Δ (KCl)	228 (175%)	35 (119%)	46 (44%)	169 (14%)	175 (75%)

**Figure 2:**
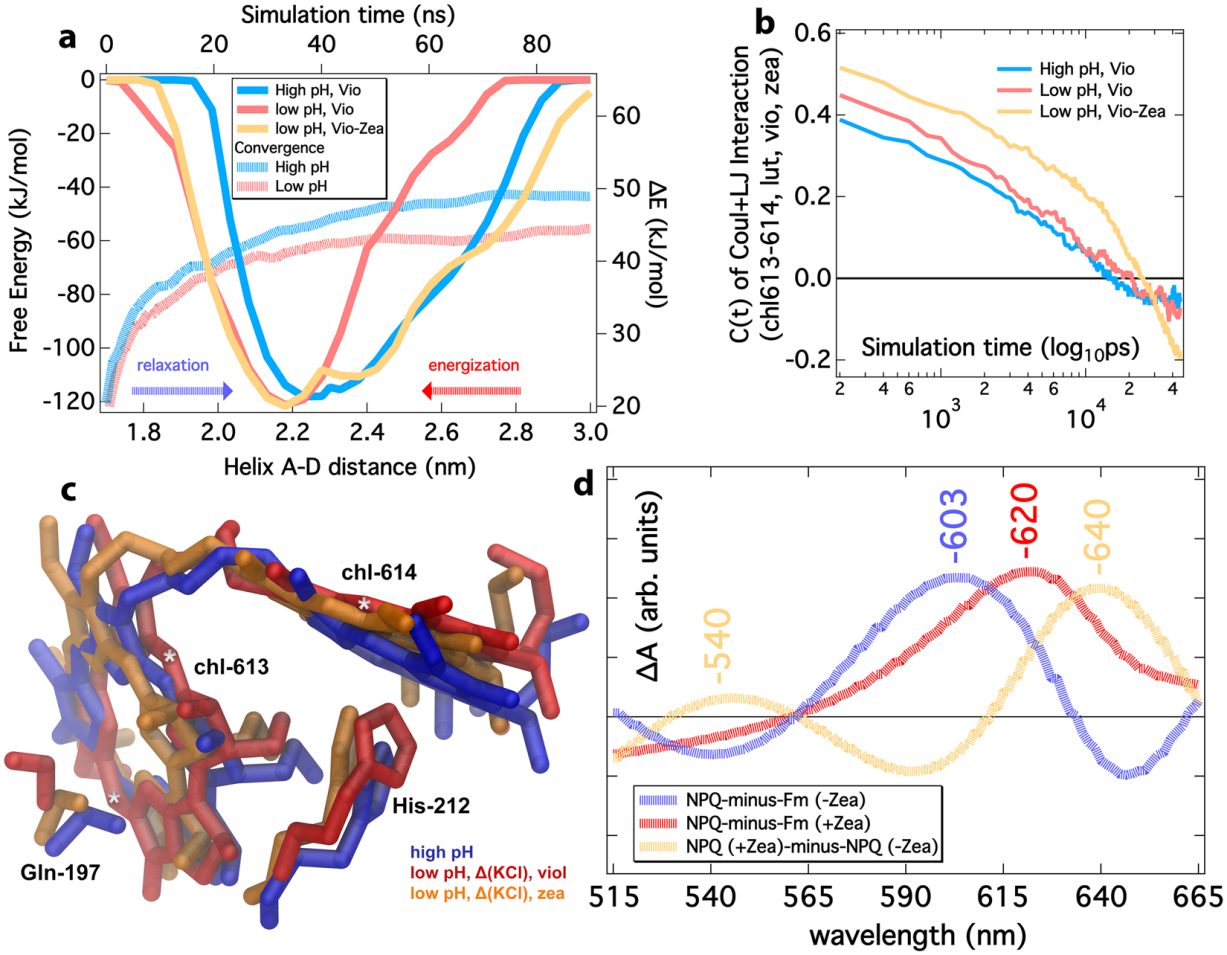
Free Energy profiles, Structures and Absorption Spectra of the Chl-613/Chl-614 pair in the major Light Harvesting Complex (LHCII) from spinach; relaxed, or under NPQ conditions. (**a**) The concatenated free energy profiles (left-bottom axes) for the major LHCII from spinach, at different lumen states – high pH (0.0/0.5 M Δ(KCl)) and low pH (0.0/0.5 M Δ(KCl) – and the violaxanthin (Vio) – Zeaxanthin (Zea) replacement effect. The average ΔΕ convergence (right-top axes) is also shown over the simulation time selectively for low and high pH between two regions (2.0–2.5 nm/2.8–3.0 nm) of the Free Energy profiles (dashed red-blue lines). (**b**) Autocorrelation Functions C(t) of the sum of Coulomb (Coul) and van der Waals (LJ) interaction energies between the pigments next to helix-D: chl613, chl614, violaxanthin (Vio), or zeaxanthin (Zea), and lutein 1 (Lut1). (**c**) *Ab-Initio* geometry optimized ground-state structures of the two pigments adjacent to helix-D (chl-613/614) from different lumen states: high pH with Violaxanthin (Vio), and low pH with Vio or Zeaxanthin (Zea). The axial ligands are treated also at the *ab initio* level (His-212 for chl614 and Gln-197 for chl613). (**d**) Relative Absorption Difference Spectra calculated at the TDDFT level with Fourier Transform of the dipoles after the electric field perturbation is applied to the Kohn-Sham states in x, y and z directions and Lorenzian fit (convolution) of the peaks. The difference absorption spectra of the chl-613/614 pair orientations are shown as NPQ-minus-Fm (−Zea) for low pH (Vio)-minus-high pH (Vio), as NPQ-minus-Fm (+Zea) for low pH (Zea)-minus-high pH (Vio) and NPQ (+zea)-minus-NPQ (−Zea) for low pH (Zea)-minus-low pH (Vio).

The values are based on the linear fit of the Mean Square Displacement (MSD) profiles versus time (t) in Fig. 2S (in SI) and the Einstein relation MSD = 6 * D * t + c, where “c” is a constant. The % changes in D_C_ values are also shown in parentheses based on the lowest energization sample (termed Ref).

From Table 1 we note that, under a ΔpH, considerably higher D_C_ coefficients are observed for all helix-pairs. For the A-D pair, there is a correlation between increasing energization (+ΔKCl → +ΔpH → +ΔpH + ΔKCl) and increasing Dc values. This means that the A-D motion is clearly pH/potential (Δ*Ψ*, Δ(KCl)) dependent, whereas B-E, C-E relative motions are less pH-dependent (high Dc values are also calculated for the high pH samples, Table 1). This does not however exclude helix-E as a secondary response site under NPQ conditions^43, 44^. Due to the distinct and conserved – among all three LHC proteins probed herein – helix-D mobility, under NPQ conditions, we further probe the residues linking the A-D helices (i.e. the connecting loop) in terms of the decay of the internal rotational correlation functions of their backbones upon the lumen perturbation (*see* SI). The rotational correlation functions RCF_rot_(t) are shown in Fig. 1f. The RCF_rot_ measures the backbone relaxation to a new conformation (equilibrium state) over time^45^. In our case, we analyze the local orientation of a segment of the backbone transitioning from lower to higher ΔpH/Δ(KCl) states. Information on the mechanism of rotational diffusion of the local probes (dihedrals) can be extracted at the short time scale. In the inset of Fig. 1f, the RCFs_rot_ are accurately fitted by single exponential decay functions. This is true for all LHCs proteins probed in this way (with wt Cars), indicating another common feature. The grouping of lower/higher lumen pH samples independently to the presence of Δ(KCl), is another indication that Cars stabilize the proteins in excess membrane energizations. The exponential decay for the RCF_rot_ exerts relaxation times t_f_ at 4.1μs (higher lumen pH) and at 2.2μs (lower lumen pH), based on the RCF_rot_(t) = a  * exp(−t/t_f_) fitting^45^. This indicates a two-fold acceleration of the helix-D motion under NPQ conditions.

### Free energy profiles

Energetics at the empirical or quantum levels are better descriptors of the LHC response upon ΔpH, Δ(KCl) or when the xanthophyll cycle is activated and the conversion of Vio to Zea takes place^13^. Due to the highly expensive computational methodologies that one needs to employ for the energetics (metadynamics and *ab initio* simulations), we have chosen to further probe only the major LHCII from spinach, in the presence of Cars, given also the fact that all LHCs studied herein exert a common response to ΔpH, Δ(KCl) in the presence of Cars. In addition, the efficiency of the Vio de-epoxidation was found to be the highest in the major LHCII, while Vio is almost inaccessible and strongly bound in CP29^46, 47^.

We have calculated the free energy profiles of the helix-D L-M motion employing the widely used and established Well-Tempered Metadynamics method (WT-MetaD)^48–50^, at the isothermal-isobaric (NpT, 310 K, 1 atm) ensemble. Figure 2a shows the concatenated free energy profiles at high lumen pH (0.0–0.5 M KCl), or low lumen pH (0.0–0.5 M KCl) in the presence of Vio (wt-LHCs), or Zea (Vio−Zea replacement). The convergence of the free energy profiles is also shown selectively for the high/low pH cases, as these profiles show the most pronounced differences in the location of the minima in respect to helix A-D distance. As a general trend, the samples at lower membrane energization (higher lumen pH) tend to exhibit lower energies at higher helix A-D distances, whereas the samples at higher membrane energization (lower lumen pH), exhibit similar free energy profiles, but with minima at lower helix A-D distances. The shift to lower helix A-D distances accompanies lower pH lumen states, in line with a recent hypothesis and an all atom study of CP29^27, 28^. A remarkable difference is exhibited for the Zea enriched major LHCII for the low pH samples. The related Free Energy profile seems to provide a link between the higher and lower A-D distances, or lower and higher membrane energizations, respectively, taking a broader shape. It is noteworthy that the relaxation from the lower lumen pH to the higher pH free energy profiles in the presence of Zea is accompanied by an energy barrier, whereas the transition from lower to higher energization is started even at higher helix A-D distances in the Zea enriched samples. The average autocorrelation function C(t) of the sum of Coulombic (Coul) and van der Waals (LJ) interaction energies between the pigments adjacent to helix-D in the major LHCII from spinach (chl613, chl614, Vio–wt, or Zea forms) is depicted in Fig. 2b at different membrane energizations. It represents the relaxation of their interaction energy, upon the employment of the Well-Tempered metadynamics bias potential. The slower relaxation can be interpreted as a closer packing of the pigments next to helix-D (and thus slowly decaying interaction energies) that is triggered upon lowering the pH at the lumen side or in the presence of Zea (red and orange solid lines, Fig. 2b). We note that the relaxation is slower in the presence of Zea, and faster at higher pH lumen states. An intermediate relaxation profile is exerted at lower pH lumen states in the presence of Vio. The aforementioned closer packing, or the strengthening of the interactions between Chl-Chl and/or Chl-Car sites has been proposed to lead to quenching^11, 24, 25, 51, 52^. In the presence of Zea, this interaction energy decay slows down further, implying an even closer pigment packing adjacent to helix-D, in line with experimental evidence that Zea presence in LHCII controls the amplitude and relaxation of quenching^13^, and increases the pigment-pigment coupling in conditions mimicking high light intensity^11^. Closer packing means steric contacts that can be important also for cooling a vibrationally excited Chl during photochemical quenching. In other words, heat dissipation by Chl can increase vibrations, whereas steric contacts may impede vibrations.

Up to this point we have only shown that several features of NPQ correlate well with our observations related to the helix-D motion, upon thylakoid membrane energization. In line with the experimental literature we observe that the effects from low pH and Zea are additive (Table 1, A–D helices)^53^. Most likely this is done by the increase in hydrophobicity of the region close to helix-D (Vio → Zea replacement), that attracts helix-D towards the thylakoid membrane, enforcing a closer packing of the adjacent pigments^13^. The region around Zea has been also proposed to relate with quenching^53^. This comes also as a validation of the proposed role for helix-D, which supports the notion that qE is allowed to build up at sub-saturating levels of ΔpH^13^, which are associated with larger helix A-D distances proposed herein. Intermediate levels of energization, which in turn lead to intermediate distance of A-D helices, allow moderate level of pigment packing and thus sub-saturating levels of quenching. This plasticity is a key feature of qE and important for the adaptation of the organism. The energy barrier in the Zea-enriched state (Fig. 2a) is in agreement with experimental observations^8–10, 13^ of a decelerated qE relaxation in the presence of Zea (Fig. 2b). Due to the overlapping of the free energy profiles in the presence of Zea at lower and higher lumen pH (Fig. 2a) we expect an accelerated qE formation even at higher pH values, associated with higher helix A-D distances. The aforementioned findings might not be sufficient to support a quenching role for the domain containing helix-D and the adjacent pigments, but provide a first evidence. To narrow such ambiguity, we calculate the *ab initio* excitation/absorption spectra of the chl-613/chl-614 pigment pair, extracted at the metadynamics free energy minima (Fig. 2a), from the different lumen states and carotenoid composition (Vio vs. Zea). This Chl pair is adjacent to helix-D.

### Chl 613/614 pair

The structures of the chl-613/chl-614 pigment pair (major LHCII from spinach) extracted at the free energy minima of different lumen states and Car composition (Fig. 2a) are optimized at the *ab initio level* (Fig. 2c). We note that despite the similar optimized His212-Mg^chl614^ distances at 0.229 nm for all species, the low pH structures appear to adopt a more domed macrocycle (Fig. 2c, red and orange structures). The dihedral angles between the two Chl planes, measured as the angles defined by CHD-CHB^chl-613^-CHD-CHB^chl-614^ atoms, are optimized at -92.3 degrees (higher pH), to -85.7 (lower pH, Vio) and up to -81.4 (lower pH, Zea) degrees. Selected CHD/CHB atoms are designated by an asterisk ( * ) in Fig. 2c for Chl-613/614 and refer to the only carbon atoms in the Chl macrocycles directly bonded to hydrogens. A change in the pigment orientation has been associated previously with a change in light-harvesting efficiency of the bacteriochlorophylls (Bchl) pigments in the purple bacteria photosynthetic apparatus^54^.

### Excitation and Absorption Spectra

The spectra of Chls exert four bands; at lower energies lie the Q_y_ and Q_x_ bands, and at higher energies the B_x_ and B_y_ bands (Soret). We focus our interest to the lowest energy Qy exciton state only, as NPQ is related with energy removal from the Qy electronic excitations^31, 55, 56^. In addition, due to the relatively large size of the *ab-initio* system (pair of chlorophylls) there might be convergence issues of the Soret that involves mixing from higher energy states (only twenty states were used due to limitations in the method employed). The electronic excitation spectral characteristics of isolated chlorophyll pigments exert peaks between 1.9–2.0 eV for the Q-band in a recent TD-DFT computational study^29^, while the experimental values in LHCII appear at 1.86 eV for the Q-band^57^. Higher energies are expected for in *vacuo* simulated spectra, as *in vacuo* experiments demonstrate a shift of the Q-band to 1.93 eV (chl-a) and 1.98 eV (chl-b)^58, 59^. In our study, following the ground-state geometry optimizations of the Chl 613/614 pairs at different conformations, their electronic excitation spectra (**Qy**) were derived (Table 2). The excitation spectra of an isolated Chl-614 (Chl-a) pigment was also calculated at the same level of theory for comparison. Our simulation of the excitation spectra in the Qy region (Table 2) for the isolated Chl-a (Chl-614), with peak at 2.042 eV shows a very good agreement with the literature. Deviations from the experimental spectra are accepted, while the general trends in energy are maintained^31^. This might be due to the absence of nearby pigments, or components of the protein matrix interacting with the Chls. In the transition from high to low lumen pH, for the Chl-a pairs, we clearly observe (Table 2) redshifts of the Qy-bands (down to ~1.4 eV). This can be associated with energy removal from the Qy band that might relate to NPQ.

**Table 2:** Vertical excitation energies (**Qy band**) of an isolated Chl-a and the chl-613/614 pigment pair ground state (in eV) at different lumen states in the presence of Violaxanthin (Vio), or Zeaxanthin (Zea).

Absorption	Excitation	single chl-a	High pH, Vio chl-a pair	Low pH, Vio chl-a pair	Low pH, Zea chl-a pair
**Qy band**	HOMO → LUMO [2 2 0 0] → [2 1 1 0]	**2**.**042**	**1**.**515–1**.**500**	**1**.**606–1**.**521–1452–1414**	**1**.**424**

We can consider two states of the LHCII: (a) the efficient light harvesting (termed Fm^55^) and (b) the photoprotective state (termed NPQ). Based on our systems (Fig. 2c), we could make a rough assignment for the high pH (Vio) Chl pair conformation as the Fm state, the low pH (Vio) conformation as the NPQ (−Zea) state, and the low pH (Vio−Zea conversion) conformation as the NPQ (+Zea) one. We note that in the *ab initio* calculations no carotenoid has been introduced in the quantum system, however the effect of the carotenoid presence (Vio, Zea) is expressed indirectly, as inducing different Chl 613/614 packing (Fig. 2c). Thus, these *ab initio* models should be considered as a simple approach for Fm-like, or NPQ-like states and not the actual Fm/NPQ states *in vivo* that include contributions from other species, as well. The relative absorption difference (ΔA) spectra of the NPQ–*minus*–Fm states in the presence (+Zea), or absence of Zea (−Zea) are shown as dashed lines in Fig. 2d. We observe similar absorption redshift at this region for the NPQ-minus-Fm states and also a redshift of the ΔΑ peak at the +Zea state (compared to −Zea) at low pH (603 → 620 nm). This comes in qualitative agreement with experimental evidence on the +Zea effect on NPQ-minus-Fm absorption difference spectra^55^. The larger redshifts calculated herein, compared to the experimental picture, could be the result of the pair isolation, or system setup, and the absence of the interacting environment (protein matrix, carotenoids). The changes in the Qy region could be justified by the change in the Chl orientations within the pair upon protein conformational changes (pH, and Zea induced). In fact, the change in the orientation of Chls in a pair can affect the light harvesting efficiency^54^. Based on these *ab initio* simulations, we can only identify redshifts in the Qy bands due to the change in the orientation of the Chls within the 613/614 pair. These changes could be related to red emitting states^60^, or to the experimentally observed changes at the 420–700 nm region^9, 61^, in the transition from a light harvesting to a quenching (NPQ) state, reported in the literature. However, computationally expensive *ab initio* Molecular Dynamics need to be employed and provide non-radiative decay rates and populations of electronically excited states or identify physical states and a direct connection with NPQ^31^. At this stage, this falls out of the scope of this study, especially for the considerably large quantum system we treated at the TDDFT level and will be the focus of future research.

### LHCII in larger membrane patch

**Figure 3:**
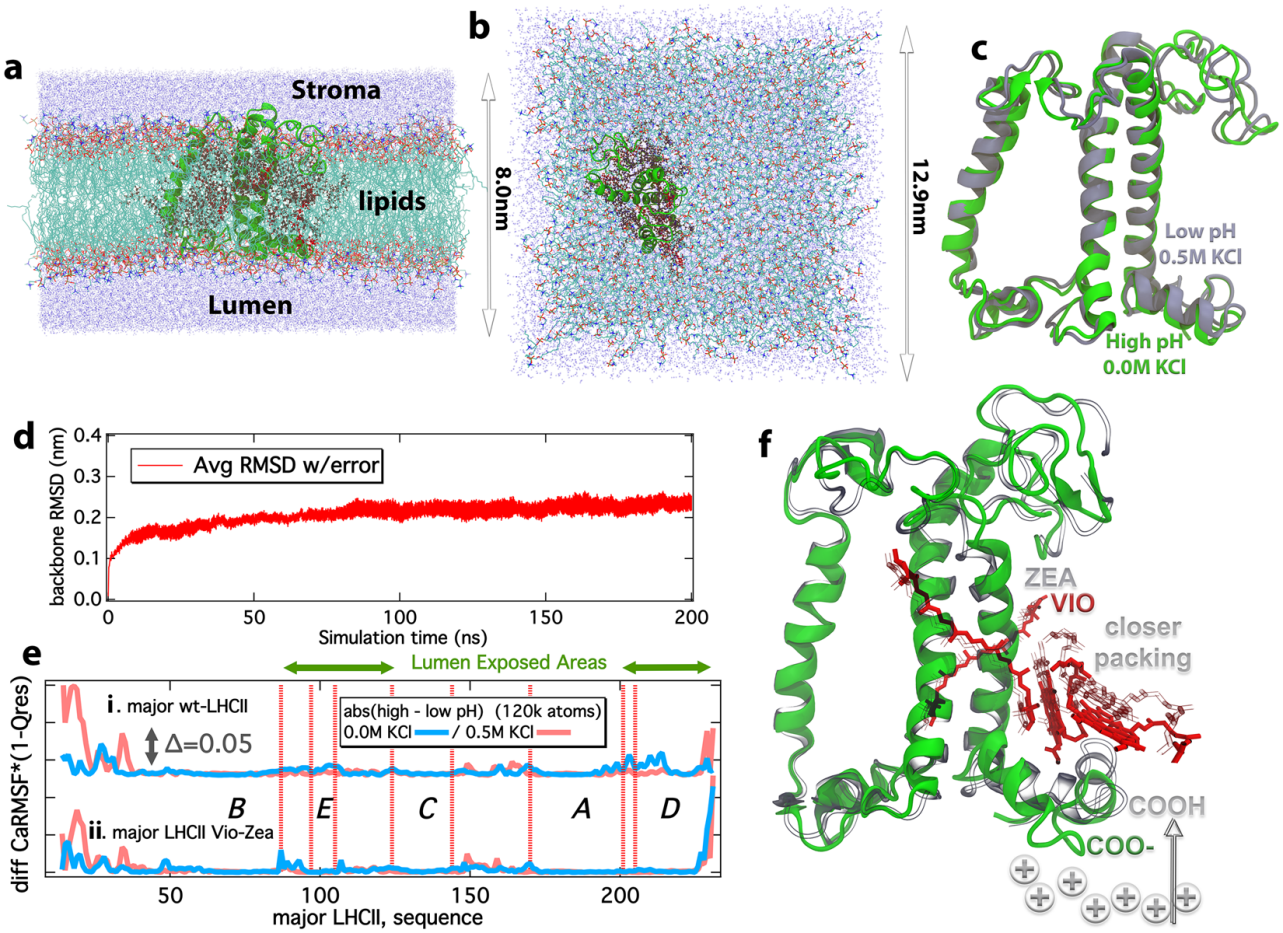
Simulations employing large membrane patches and a proposed model for the response of LHCs under NPQ conditions. (**a**,**b**) Side and Top views of the large membrane systems, with dimensions indicated. Water is shown in blue dots, lipid tails in light gray and lipid heads in red (oxygen) – blue (nitrogen). The protein matrix is shown in green cartoons, carotenoids in red and chlorophylls in dark gray. (**c**) Average structures out of the cluster averaging (400 ns per sample) for the high pH (0.0 M KCl) (green) and low pH (0.5 M KCl) (ice blue) states. (**d**) The average backbone Root mean Square Deviation (RMSD) in nm for all major LHCII (spinach) large membrane structures (400 ns cumulative sampling time per sample). (**e**) The product of Ca-RMSF * (1-Qres) in absolute high minus low pH difference in the presence (red) or in the absence (blue) of a Δ(KCl). The peaks indicate regions of high flexibility and distinct structural conformation for the major LHCII (spinach) in a large membrane patch and 400 ns cumulative sampling time per sample. (**f**) Schematic overview of the proposed response of the antenna complexes of Photosystem II, under NPQ conditions. Solid colors represent relaxing states (light harvesting), whereas transparent/gray colors represent a potential energy dissipating state (quenching), based on the major LHCII (spinach) response to ΔpH/Δ(KCl). Different scaling has been used for the cartoons (green, helices-residues) and the pigments (red) for clarity.

The Molecular Dynamics results presented so far are based on systems containing around 40000 atoms, including the LHCII complexes, a membrane patch of between 120–128 united-atom UA-POPC lipids and 8500 TIP3P^33^ water molecules at 6.5 × 6.5 nm^2^ lateral dimensions. A liquid-vapor interface for the water pools on either side of the membrane was introduced in the system setup, and constraints have been applied to the movement of lipids (see SI). Despite the fact that the employed number of lipids is quite common in membrane simulations, balancing between accuracy and efficiency of simulated properties^62^, we have embedded the major LHCII (spinach) with all pigments (Chls, Cars) in a larger membrane patch that includes between 480–490 united-atom UA-POPC lipids^63, 64^, along with around 22200 TIP4P waters (Fig. 3a,b)^65^. This raised the number of simulated atoms roughly 3-fold, to 120000. The major LHCII from spinach has been prepared accordingly at low and high pH^28^, at 0.0 and 0.5 M KCl concentrations (six samples, including two with a Vio−Zea conversion at low pH). No liquid-vapor interface (vacuum) has been introduced, nor constraints in the lipid movement are enforced for these larger systems. The lateral dimensions of the new (larger) systems are 12.9 × 12.9 nm^2^, whereas the dimension on the axis perpendicular to the membrane plane is at 9.0 nm (Fig. 3a,b). The samples were simulated with the same protocol as the smaller systems (equilibration, production, *refer to the methods section and the SI*), with the exception that the production simulations were extended from 90 ns to 200 ns in two independent trajectories per sample (400 ns in total per sample). This simulation time is common in integrated large proteins in all-atom CMD simulations^66^. These larger systems serve to probe potential artifacts in the smaller systems arising from (i) finite system size effects including spurious interactions between periodic images, (ii) the constraints enforced for the C50 atom in the POPC lipids (see SI), (iii) the choice of TIP3P over TIP4P water model, and (iv) the vacuum needed to generate a ΔΨ gradient in the membrane that introduces a liquid-vapor interface, but also a well-defined water bulk phase that interacts with the LHCII in the system (refer also to the SI, Fig. 1S in SI). The average structures out of the CMD trajectories for the high pH, 0.0 M KCl (lower energization, green) and the low pH, 0.5 M KCl (higher energization, ice blue) are shown in a superposition scheme in Fig. 3c. If compared with the structures in Fig. 1c, we observe an identical trend in the context of the L-M conformational change proposed based on the dynamics in the smaller systems. We have to note that the analysis on the production trajectories has been performed after the first 40 ns, where the backbone LHCII RMSD value has reached a plateau (Fig. 3d) for the larger systems. In addition, the calculated Ca-RMSF * (1-Qres) peaks for the larger systems and the major LHCII (spinach) in Fig. 3e are in agreement with the dynamics calculated for the smaller systems and constrained lipids previously presented (Fig. 1e), with dominant peaks at the helix-D region. Zea presence, enhances the LHCII response to *pmf* even at lower energizations (ΔpH only). However, the simulations with all-atom larger systems prove inefficient in terms of computational resources, while the smaller systems we have employed extensively in this study, provide the same insight, without obvious artifacts due to their smaller size. Especially for the Well-Tempered Metadyanamics, the large systems required considerably higher simulation sampling (slow convergence) and computational time, so we had to make a choice between system size and computational efficiency. This however does not compromise the results, or conclusions in this study.

## Discussion

The molecular (meta) dynamics simulations uncover a critical role played by the helix-D of the LHCII in governing the intramolecular packing of the adjacent pigments with consequences on the excitation energy transfer, in line with recent proposals^27, 28^. In summary, for the first time we link ΔpH/ΔΨ to conformational changes at the atomic level in the LHCs (major LHCII, minor CP29) that affect excitation/absorption spectra of pigments within the LHCs (Qy band), under NPQ conditions (i.e. high membrane energization). This describes a new pathway that could lead to a new quenching site (Chl613-614 pair) in terms of Non-Photochemical Quenching. This pathway is present in all LHCs probed herein. The principle aspects of the proposed mechanism include a ΔpH that protonates carboxyl groups at the lumen side (Fig. 3f), enabling their accommodation as neutral moieties closer to the membrane lipids. This induces conformational changes in the helix–D environment that leads to closer pigment packing in the vicinity. In line with experimental evidence that the Vio−Zea conversion in LHCII does not lead to quenching^67^, Zea presence can render the related site more hydrophobic, enhancing the latter closer packing (Fig. 3f) and induce redshift in absorption spectra. Vio−Zea conversion could make the potential energy trap more efficient, more stable and with slower relaxation. In any case, our results, supporting also previous proposals^13^, show that Zea presence is not required for NPQ, nor does it trigger NPQ, but can enhance it. The simplified model we propose does not take into account other numerous components that can affect NPQ (like the PsbS protein^68^), but our pathway could be an additional component of the LHCs response under NPQ conditions. In contrast to other models, this model can produce intermediate levels of quenching upon moderate changes of lumen pH, which in turn produce moderate conformational changes, exerted as different helix A-D distances in LHCs.

It seems likely that Photosystem II has developed numerous photoprotective mechanisms throughout its evolution^69, 70^, several parts of which can be considered today as evolutionary leftovers, that co-work in an extended photoprotective mechanism. We expect that the helix-D plasticity proposed herein serves as such due to excimer formation between Chl-614/Chl-613. Apart from helix-D, other regions of the LHCs show a milder response under ΔpH (e.g. helix-E) and future computational studies could also focus on these regions and the adjacent pigments. The computational approach developed herein can be employed and extended in the future to probe different LHC regions, pairs of pigments, either homodimers (Chl-Chl), or heterodimers (Chl-Car) under NPQ conditions. Our findings provide a useful target for future experimental studies based on mutated residues, especially for the promising technique of single-molecule spectroscopy^71^. To test the validity of the current proposal, it is worth investigating mutants/deletions of the lumen exposed helix-D and characterize spectroscopically the response in different thylakoid membrane energizations.

## Materials and Methods

In this section, we provide a short description of the samples prepared and the methodologies employed in this study, in order to help the reader follow the results and conclusions of this study. Detailed descriptions can be found in the *Computational Details* section in the Supplementary Information (SI).

### Sample preparation

The major LHCII is found in trimeric or monomeric forms in the thylakoid membranes, and shares common structural features with the minor monomeric antenna complexes, that are able to switch between light-harvesting and photoprotective states^72–74^. Thus, in this study, we have probed only the monomeric forms of the major LHCII, for the computational efficiency and the direct comparison with the minor LHC (CP29) from spinach, which appears as a monomer. However, the results from the monomer LHCII form are transferrable to the trimeric case, in accordance with a previous study^16^. This is why we have retained all the pigment content and crystallographic waters from the trimers to the monomeric form of the major LHCII systems. The crystal structures for the minor LHC (CP29) from spinach^75^, and the major LHCII from spinach^2^ and pea^76^ were obtained from the Protein Data Bank (codes 3PL9, 1RWT and 2BHW respectively). Two protonation states were chosen to describe adequately the physiological pH range at the lumen side, termed: low pH < 6 and high pH > 6, with a ±1 tolerance and they were associated with higher and lower membrane energizations, respectively^28^. The protonation state of Glu (E) and Asp (D) residues in the thylakoid lumen side of the protein was chosen to qualitatively simulate these different pH values (see also the SI). The choice of protonations were based on previous studies that identify lumen exposed amino acids that are potentially pH sensitive^28, 43, 44^. We prepared membrane–embedded forms of the LHCs (united-atom UA-POPC lipids)^63, 64^ with a salt gradient Δ(KCl) at 0.0 or 0.5 M between stroma-lumen^28^. To achieve the 0.5 M gradient, models were enriched in K^+^, Cl^−^ ions added only to the water pool at the lumen side at a concentration of 0.5 M in line with a previous study^28^. All samples were neutralized by adding K^+^/Cl^−^ when needed (additionally to the Δ(KCl) gradient). These neutralizing counter ions are not contributing to the gradient absolute values reported herein (0.0–0.5 M), as they are only few (4–10) K^+^/Cl^−^ atoms per 8500 water molecules. The pKa values of lumen exposed Asp/Glu residues are expected to vary significantly in proteins *in vivo* with the environment or the presence of counter ions (0.5 to 9.2 for Asp and 2.1 to 8.8 for Glu)^77^. Thus, the exact *in vivo* values cannot be easily determined^78^. This is also quite difficult to simulate, especially in the case where (i) different parts of the protein sense a solvent-buffer of different pH (lumen-stroma) and (ii) we introduce the Δ(KCl) gradient, where counter ion concentration is significant in some samples and theoretical titration methods would be difficult to converge. Based on a lack of knowledge on the exact pH value that leads to protonation of each residue and the debate in the literature over the actual lumen pH value (moderate ~5.8 and 6.5, or strongly acidic at pH < 5)^79^, we have chosen to probe possible *in vivo* states associated with crucial Asp/Glu residues^28, 43, 44^ that are treated deprotonated for the high pH samples and protonated for the low pH samples. This overcomes the aforementioned drawback. Although a pH range and not a specific value is simulated in this way, the results should also be related with the *in vivo* high/low pH lumen states^28^. We have also to note that the DPPG lipid found in the crystal structures of the LHCs is a peripherally interacting lipid^2, 75, 76^ and its role can be simulated by the POPC lipids in the membrane patch employed. Thus, in the simulated systems, we have removed these DPPG lipids (two or less per structure). In the crystal structures, the DPPG lipids are away from helix-D^2, 75, 76^, or the Vio−Zea conversion site, so the results in this study should not be affected by this removal.

Apart from the wild-type (wt) LHCs based on the crystal structures, we prepared also carotenoid-deprived (without Cars) samples for all LHCs. Simulations in the absence of carotenoid molecules were performed under the same preparation protocol described for the carotenoid-rich samples. Thus, twenty-four (24) samples/starting structures were generated (3 LHCs × 4 lumen states × 2 with and without car). In addition, structures with a Violaxanthin (Vio)−Zeaxanthin (Zea) conversion in the major LHCII from spinach were studied for the low lumen pH cases only. Hence, two (2) samples containing Zea in the place of Vio in the major LHCII from spinach were added to the sample pool. Vio de-epoxidase is activated under low lumen pH, and it is found to exert high efficiency in the major LHCII, wheares Vio is almost inaccessible in CP29^46, 47^, which justifies our choice. Its exact place, though, within the LHCII, is not entirely clear and currently under debate^7^, that is why we have chosen to replace Vio by Zea, which occurs under stress (NPQ)^13, 34^, as a first approach. Further studies will address different binding sites of Zea, proposed in the literature (like the L2 site within LHCII, or L1 within Lhcb5)^34, 36^.

### Classical Molecular Dynamics and Metadynamics Simulations

The twenty-six (26) aforementioned starting structures at the all-atom resolution were fed to CMD production trajectories. The 0.5 M concentration of salt, employed for some samples, is slightly higher than the physiological range, but it is a common practice in CMD to exaggerate on concentrations in order for the effects to show in a reasonable simulation time and achieve an efficient phase space sampling. The CMD trajectories were propagated for a total time of around 13μs. CMD simulations were followed by Well-Tempered Metadynamics runs^48–50^, to describe free energy profiles of the conformational changes occurring in the major LHCII from spinach, under the NPQ conditions. All the simulations were performed at the isothermal-isobaric (NpT, 310 K, 1 atm) ensemble, with a cumulative simulation time of 15 μs. For all CMD and metadynamics, three independent (different starting structures at the end of the equilibration-relaxation stage) production trajectories of 90 ns were run per sample and the results were averaged. In the Metadynamics simulation scheme, a history-dependent biasing potential is constructed on the fly as a function of Collective Variables (CV) of the system. The fluctuations in the CVs are gradually enhanced and the system is discouraged from visiting previous configurations, escaping from the low free-energy basins of the phase space towards an efficient phase-space sampling. The distance between the center of masses of the A-D helices was used as a single CV in this case.

### *Ab Initio* Simulations

The *ab-initio* simulations were based on the time-dependent DFT (TDDFT) method^35^ with plane wave framework. Initial structures (chl-613/614 pair including the axial ligands) were extracted at the free energy minima, out of the Well-Tempered metadynamics runs. We calculated ground state geometries and electronic excitation/absorption spectra of the Chl pigments of the major LHCII from spinach, under the different lumen states and pigment content. Different functionals (BLYP, revPBE, PW91) were tested for the calculations, but we faced several convergence or accuracy issues. The combination of the PBE Density Functional^80, 81^ in a plane-wave pseudopotential norm-conserving (NCPP) framework available in the MT (Troullier-Martins) scheme^82^ and the Kleinman-Bylander approximation for the effect of core electrons^83^, proved ideal, as it is also employed elsewhere and has shown remarkable accuracy for porphyrins and chlorophylls alike^32, 84, 85^.

### Data Availability

All data generated or analyzed during this study are presented in this published article (and its Supplementary Information files) and are freely available to readers. Correspondence and requests for materials should be addressed to V.D. (evangelos.daskalakis@cut.ac.cy).

## Electronic supplementary material

Supplementary Information
