# Supplementary material for: A pathway for protective quenching in antenna proteins of Photosystem II
Source: Sci Rep. 2017 May 31;7:2523. doi: 10.1038/s41598-017-02892-w (PMC5451436; doi:10.1038/s41598-017-02892-w)
Supplement: Supplementary file 1 — Supplementary Information [file 41598_2017_2892_MOESM1_ESM.pdf]

A pathway for protective quenching in antenna proteins of Photosystem II

Sotiris Papadatos, Antreas C. Charalambous, and Vangelis Daskalakis*

Department of Environmental Science and Technology, Cyprus University of Technology, 30 Archbishop Kyprianou Str., 3603 Limassol, Cyprus

*Corresponding Author

e-mail: evangelos.daskalakis@cut.ac.cy

Tel: +357 25002458/ Fax: +357 25002820

Contents

1. Computational Details

2. The Stabilizing Effect of Carotenoids

3. RMSF and Qres Values

4. On the fly perturbation of the lumen buffer

1. Computational Details

Sample Preparation. The major LHCII from spinach (pdb code: 1RWT)[1] and pea (pdb code: 2BHW)[2] contain 14 chlorophyll (Chl) pigments and 4 carotenoids (Cars) each (see **Figure 1a** in the *main manuscript* for the structure of the major LHCII from spinach). Four Chl pigments in 1RWT (605, 606, 604 and 614) and five Chls in 2BHW (613, 614, 602, 606 and 608) were incomplete in the resolved structures, with missing carbon atoms in the phytol chains, resulting in partial sets of coordinates. The missing atoms were substituted based on the corresponding Chl pigments in CP29 (pdb code: 3PL9)[3] after superpositioning the 1RWT-3PL9 and 2BHW-3PL9 resolved structures, using structural alignment in MultiSeq of VMD software.[4,5] This choice is based on the fact that Chl pigments in CP29[3] are completely resolved, contrary to the major LHCII structures resolved from spinach and pea employed.[1,2] In the CP29 structure used, only heteroatoms from the HTG (heptyl 1-thiohexopyranoside) are not resolved, but this ligand is not simulated in this study. All crystallographic water molecules were also retained. We provide **Table 1S**, with the detailed substitutions in pairs of Chl pigments from 3PL9 to 1RWT and 2BHW structures. In the cases where Chl-a matched with a Chl-b (Chl-a 608 from 2BHW and Chl-a 614 from 1RWT with Chl-b 614 in 3PL9), a formyl oxygen was removed to match the Chl species. In mismatched Chl pigments between 1RWT (Chl-b 605) or 2BHW (Chl-b 614) and the 3PL9 structures, the phytol chains were re-generated by using an arbitrary Chl group within the same structure, by superimposing the Chl pigments on the magnesium and adjacent nitrogen atoms.

To simulate lower pH values at the lumen, specific Glu (E) and Asp (D) residues were protonated at the lumen side, as shown in **Table 2S** for the different Light Harvesting Complexes (LHCs). The latter protonations are based on previous studies that identify lumen exposed amino acids that are potentially pH sensitive.[6-8] The rest of Glu, Asp residues in the protein were left deprotonated. All His residues were protonated at the N_δ sites, after visual inspection that ensures that the site of protonation

(N_δ/ N_ε) does not disturb the crystal structure, based on the formation of inter-residue hydrogen bonds.[9] Especially for CP29, E151 is not conserved among the major LHCII and it is found in the trans-membrane space of the protein. E151 was kept protonated, in line with a recent study [8] for all samples. All systems are neutralized by adding ions (K^+/ Cl^-), if needed, in any case.

3PL9	1RWT	2BHW
chl-b 606	chl-b 606	chl-b 613
chl-b 614	chl-a 614	chl-a 608
n/a	chl-b 605	chl-b 614
chl-a 612	n/a	chl-a 602
chl-a 604	chl-a 604	chl-a 606

Table 1S. Substitutions between completely resolved Chl-a/b pigments in CP29 from spinach (pdb code: 3PL9) and major LHCII from spinach (pdb code: 1RWT) and pea (pdb code: 2BHW).

LHC	Glu	Asp
minor CP29, spinach	114, 128, 131	123, 232, 240
major LHCII, spinach	83, 94, 107, 207	111, 211, 215
major LHCII, pea	83, 94, 107, 207	111, 211,215

Table 2S. Lumen exposed residues that were kept protonated for the low pH samples and deprotonated for the high pH samples.

The models of LHCs at low and high lumen pH were embedded in a membrane lipid bilayer patch that contained around 120 POPC lipids using the g_membed method [10] with around 6.5 x 6.5 nm^2 lateral dimensions. The principal axis of each protein was aligned to the z-axis. Around 8500 TIP3P [11,12]

water molecules were used to solvate the models above or below each membrane surface, filling the z-dimension from 0 to around 10nm. No solvating waters were added to the membrane or protein area and almost equimolecular water pools were created on both sides of each membrane, keeping the water/ lipid ratio at around 69, well above the experimental ratio of 32.5.[13] The z-dimension was then extended to 25.0nm, to minimize the interaction between the water pools interacting with the two sides of each lipid bilayer membrane. This introduces a liquid-vapor interface and enables the separation of the water pools to achieve different concentration of KCl in each side of the membrane.

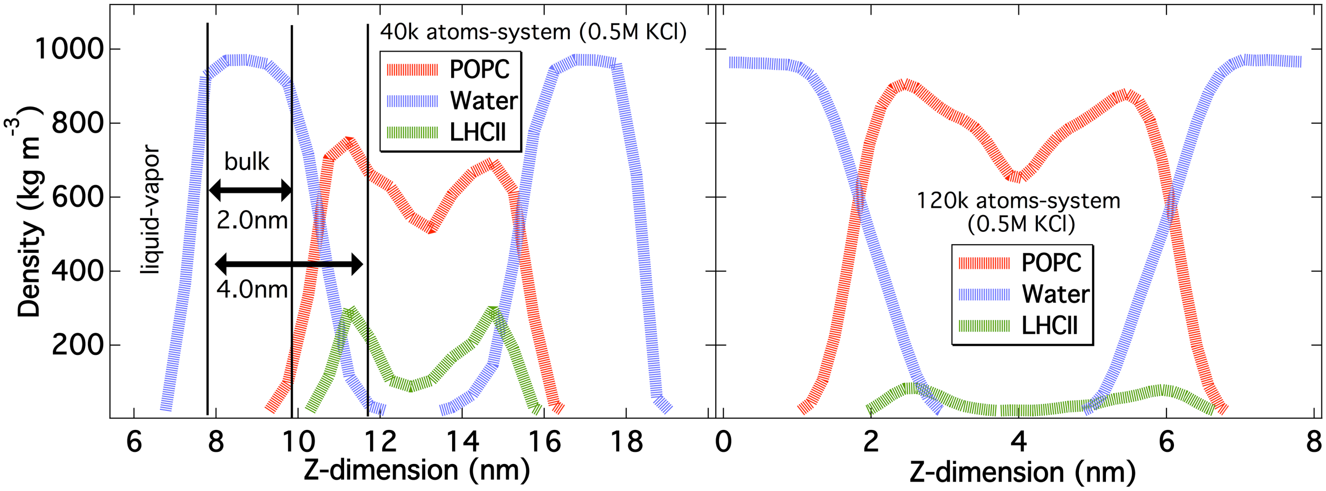

Figure 1S. The densities of the system (40k or 120k atoms) components (POPC lipids, Water, LHCII protein) against the Z-dimension (axis perpendicular to the membrane plane).

The GROMACS 5.0.7 [14] software package was employed for the Classical Molecular Dynamics (CMD) simulations. For all simulations the OPLS all-atom protein force field [15] was employed for the residues. For the chlorophyll (Chl) pigments OPLS compatible parameters were used. [16] The carotenoids were described based on the potential assigned by the swiss param database,[17] but adapted also in previous studies for compatibility with OPLS-AA and the GROMACS input,[8,18] in line with a standard parametrization protocol.[19] OPLS united-atom lipid parameters for POPC were employed, used also previously at

310K to probe membrane dynamics [20,21] along with the TIP3P water model. [11,12] Short-range Coulombic interactions were truncated at 1.2nm, whereas long-range Coulombic interactions were computed using the particle mesh Ewald (PME) method [22], with PME order of 4, Fourier spacing of 0.15 nm, and a dielectric permittivity of 1. A switched cutoff radius between 1.0−1.2 nm was used for the van der Waals interactions for compatibility with the pigment parameters [16]. Dispersion Corrections for energy and pressure were also applied due to the truncation scheme employed. Solvent (water, ions), lipids, and protein were each coupled separately to a heat bath. Restraints involving bonds of hydrogen atoms were employed using LINCS [23], while the SETTLE algorithm [24] was used to restrain water bonds with force constants of 1000 kj $mol^{-1}nm^{-2}$.

Equilibration/ Relaxation. All the heavy atoms of the protein (including pigment heavy atoms) were restrained with force constants of 1000 kj $mol^{-1}nm^{-2}$ by the LINCS [23] algorithm. All twenty-six (26) samples were minimized in energy with the steepest descend method for 2000 steps followed by constant density and temperature (NVT) Brownian dynamics (BD) (1fs time step) at 10K for 300ps employing the berendsen thermostat, with a temperature coupling constant at 1fs. [25] Then they were heated from 10K to 310K along with the gradual removal of the protein heavy-atom restraints. In detail, two short constant density (NVT) and constant pressure (NpT) runs lasted for 25ps each, with a weak coupling berendsen thermostat and barostat [25] at 10K employing time coupling constants of 0.1ps for the temperature and semiisotropic 50.0ps coupling for the pressure with a compressibility of $4.6x10^{-5}$ (x-y dimension) and 0 (z dimension) for the smaller systems (40k atoms), or $4.6x10^{-5}$ (x-y dimension) and $4.5x10^{-5}$ (z dimension) for the larger systems (120k atoms). Then, the samples were gradually heated from 10K to 250K in a constant density ensemble (NVT) for 3ns employing the v-rescale thermostat [26], with a time coupling constant of 0.1ps. The temperature was increased from 250K to 310K in a con-

stant pressure ensemble (NpT) for 2ns, employing the v-rescale thermostat [26] and berendsen barostat [25], with time coupling constants of 0.1ps for the temperature and 2.0ps for the pressure, removing also all but the Ca-atom protein restraints (LINKS). This latter was followed by an equilibration at 310K (0.1ps temperature coupling constant) for 1ns (NpT, 1atm, 2.0ps coupling constant for pressure), without any restraints for the protein heavy-atoms or the lipids. For the equilibration, all steps were integrated with a 1fs time step, except when explicitly noted otherwise.

Molecular Dynamics production trajectories. The Newton's equations of motion were integrated for 90ns in three independent trajectories per equilibrated-relaxed sample for all LHC proteins. This resulted in a total of seventy-eight (78) production trajectories (26x3 production trajectories per sample) or ~7.0 μs simulation time for the CMD runs. A second set of production runs (referred to as *on the fly* perturbation at the lumen) was initiated from all wt LHCs starting structures that were extracted at the end of the previous 90ns production trajectories of the wild-type (wt) High pH/ 0.0M KCl, termed reference sample (Ref). For this second set, the Glu-Asp protonations were changed manually to mimic the transition to a different lumen configuration *on the fly* (**Figure 1b,** *main manuscript*). When needed, 0.5M K^+ and Cl^- were also introduced at the lumen side to simulate the KCl gradient for the higher membrane energization samples, by using the 'genion' tool in GROMACS. Energy minimization and short BD runs of 1ns were employed before the production sampling in this case, to assure no large forces or close contacts are introduced by changing the state of the lumen exposed residues to their low pH protonated states (**Table 2S**). This short equilibration time was necessary in order not to lose any important *out-of-equilibrium* response due to abrupt buffer change and also eliminate any bad contacts introduced (between protons, ions, waters). We started the sampling of the conformational changes in the protein, immediately after. Again, three independent trajectories (90ns each) were run

per sample, as well as for the reference sample, practically extending the simulation time of this reference sample from 90ns to 180ns. This second set of runs was only performed for the carotenoid-rich (wt) samples, with a total production simulation time of around 3.24μs and probed the dynamics upon the perturbation in the lumen after the short equilibration.

All production simulations were run with a 2 fs integration time step with the leap-frog integrator in GROMACS 5.07.[14] They were performed at a constant pressure NpT ensemble employing the v-rescale thermostat [26] and *Parrinello-Rahman* barostat. [27,28] Solvent (water, ions), lipids, and the protein were each coupled separately to a heat bath at a temperature of 310K with time coupling constant 0.5ps. Pressure of 1 bar was maintained using semi-isotropic pressure coupling with compressibility $4.6x10^{-5}$ bar^{-1} (x-y dimension) and 0 (z dimension) for the smaller systems (40k atoms), or $4.6x10^{-5}$ (x-y dimension) and $4.5x10^{-5}$ (z dimension) for the larger systems (120k atoms), and a time coupling constant of 2ps. We have to note that for selected POPC lipids, the C50 atom (numbering from ref [20]), located at roughly the middle of each lipid, was restrained at its position after the equilibration and relaxation with a force constant of 1000 kj $mol^{-1}nm^{-2}$. This was necessary because certain lumen buffers were introducing instability of the membrane patches, in combination with the large (25nm) mostly empty spaced z dimension of the simulation unit cell. This large vacuum in z dimension is introduced to separate the water pools at either side of the membrane and it is a common approach in MD simulations that introduces a liquid-vapor interface with no artifacts in pressure.[29,30] We have also to note that despite the induction of the liquid – vapor interface in the water pools, the latter are large enough to have a well-defined bulk phase (at least 2.0nm depth), with which the protein interacts, so no side-effects from the presence of a liquid-vapor interface are expected on the protein dynamics. This is also evident from **Figure 1S**, where the densities of the different system components (POPC lipids, water and LHCII) are plotted against the Z axis (perpendicular to the membrane plane), for the typical sys-

tems employed herein. As we can observe, the protein does not interact with the liquid-vapor interface in the smaller systems (40k atoms) as it is well buried within the liquid water bulk phase/ liquid water-membrane interface. The xy lateral dimensions of the membrane, even with the C50 atoms of lipids constrained, are allowed to expand according to the pressure and compressibility with coordinate scaling employed in GROMACS.[14] This maintains the major part of membrane flexibility, and ensures stability in the setup employed. We have to note that the membrane in this case is used only to separate two water pools, to provide an hydrophobic environment for the LHCII and to simulate a ΔΨ (potential gradient) that affects the LHCII dynamics. However, no conclusions can be drawn in this case concerning membrane dynamics, as some properties of the simulated membrane could be affected.

Well-Tempered Metadynamics. The GROMACS 5.07 software patched with plumed 2.2 [31] has provided the computational engine. Three independent metadynamics trajectories for each sample and the major LHCII from spinach were run for 90ns each. Initial structures were extracted at the end (90ns) of the previous three independent CMD trajectories per sample. This totals in 20 metadynamics production trajectories (1.8μs) accounting for the wt samples in the presence of carotenoids in addition to a Vio-Zea conversion for the low pH samples. Despite the fact that the total simulation time is the same as in CMD trajectories for the major LHCII from spinach, the computational time needed was increased 4-fold per sample. The distance between the center of masses (COM) of helices A and D was set as a single collective variable (CV). The COM was calculated based on the alpha-carbon (Ca) atoms of helix A or D. In detail, these refer to the Ca atoms of the residues 205-214 (for helix-D) and 170-201 (for helix-A) for the major LHCII from spinach. A path CV is formed that involves 42 atoms for the major LHCII and the distance α (1.0 nm < α < 4.0 nm) along a reference path between states L and M (*see main manuscript*). The grid resolution was set at 0.05nm. The bias was achieved by depositing Gaussians every 1

ps, of widths that cover the space of 500 timesteps in the CV space. The starting Gaussian height was at 0.4 kJ/mol and it was gradually decreased in terms of a diffusive adaptive bias scheme with a temperature difference of ΔT=2170K (Well-Tempered metadynamics bias factor 8). This setup is based on numerous test-runs with variant values of the bias (2-12), Gaussian width (100-1000) and Height (0.2-1.4 kJ/mol) that were performed, but lead to denatured complexes, or inadequate phase space sampling.

Ab-Initio **Simulations**. Initial structures for the *ab-initio* methods on the pigment (Chl-613/ 614) ground states were extracted at the free energy minima, out of the metadynamics runs (**Figure 2a,** *main manuscript*). The quantum region contained only the two pigments, chl-613 and chl-614, adjacent to helix-D, along with their Gln-197 and His-212 axial ligands, respectively. These resulted from the empirical structures at the Free Energy minima with helix A-D distances at 2.27nm and 2.18nm for the lower (higher lumen pH) and higher (lower lumen pH) membrane energizations. The system was placed in a cubic unit cell of 4x4x4 nm^3 volume. All Chl side chains were retained, except the phytol side chains of the pigments that were truncated at the fourth carbon atom and capped with a hydrogen, as they play no important role in the absorption process.[32] The original orientations of the two pigments, based on the free energy minima from the metadynamics runs, were maintained by adding four aliphatic $–CH_2–$ groups, to covalently link the chlorophylls via aliphatic side chains, yielding a pair of pigments. This was necessary in order to effectively converge the geometry of the two pigments in the context of Density Functional Theory (DFT), maintaining the same geometry-orientation as in the respective free energy minima and avoid using position restraints that lead to imaginary frequencies. For all *ab-initio* runs, we use the PBE Density Functional[33,34] in a plane-wave pseudopotential norm-conserving (NCPP) framework. These pseudopotentials are available in the MT (Troullier-Martins) scheme[35] and can be used along with the Kleinman-Bylander approximation for the effect of core electrons.[36] Geometry optimiza-

tions for the ground state were performed with the ODIIS optimizer in CPMD 4.1 with the convergence criteria for the wavefunction and the density cutoff for the calculation of the gradient correction to be set at 1.0E-06 a.u. [37,38]. The geometry optimization criterion was set at 0.5E-04 a.u. This *ab-initio* setup has shown remarkable accuracy for porphyrins and chlorophylls alike.[39-41]

RMSD in nm	LHCII, spinach wt-Cars/ no-Cars	CP29, spinach wt-Cars/ no-Cars	LHCII, pea wt-Cars/ no-Cars	Averages wt-Cars/ no-Cars
Low pH	0.24/ 0.28	0.19/ 0.20	0.24/ 0.33	0.22/ 0.27
Low pH/ 0.5M KCl	0.23/ 0.30	0.14/ 0.26	0.33/ 0.28	0.23/ 0.28
High pH	0.29/ 0.26	0.18/ 0.23	0.20/ 0.34	0.22/ 0.28
High pH/ 0.5M KCl	0.25/ 0.24	0.22/ 0.22	0.29/ 0.26	0.25/ 0.24
Averages	0.25/ 0.27	0.18/ 0.23	0.26/ 0.30	-
**% L-M motion**	**LHCII, spinach wt-Cars/ no-Cars**	**CP29, spinach wt-Cars/ no-Cars**	**LHCII, pea wt-Cars/ no-Cars**	**Averages wt-Cars/ no-Cars**
Low pH	40.9/ 40.1	13.8/ 30.0	43.1/ 47.2	32.6/ 39.1
Low pH/ 0.5M KCl	21.4/ 57.9	21.0/ 50.0	17.2/ 51.5	19.9/ 53.1
High pH	11.5/ 46.1	30.5/ 39.8	15.2/ 20.4	19.1/ 35.4
High pH/ 0.5M KCl	14.6/ 27.4	12.5/ 52.7	28.6/ 45.3	18.6/ 41.8
Averages	22.1/ 42.9	19.4/ 43.1	26.0/ 41.1	-

Table 3S. The backbone Root Mean Square Deviation (RMSD) values in nm for the various samples, in the presence (wt-Cars)/ absence of carotenoids (no-Cars) from the CMD trajectories. The average % contribution of the L-M motion to the overall protein motion in the presence (wt-Cars)/ absence (no-Cars) of carotenoids for the same trajectories is also calculated.

The calculation of the electronic spectra was based on the time-dependent DFT (TDDFT) method[42], employed as implemented also in CPMD 4.1 in the Tamm-Dancoff approximation.[43] During the calculations, the density functionals were adopted to the local spin density (LSD) approximation to DFT. During the geometry optimization, the structures were deviating (carbon root mean square deviation – RMSD) from the original free energy minima (empirical level of theory) at an average of 0.104$\pm$0.038

nm with the final geometry to converge at an average of 0.138 nm RMSD away from the respective structures at the free energy minima at the empirical level of theory. For the absorption spectra, an instantaneous electric field perturbation is applied to the Kohn-Sham states in x, y and z directions to introduce a small momentum to the electrons and then the states are propagated with a time step of 0.003 ħ/eV. The time-propagation after the perturbation in TDDFT is continued up to 60 ħ/eV at the Cayley scheme[38] to obtain a good resolution for the Qy band.[32]

2. The Stabilizing Effect of Carotenoids

The maximum backbone Root Mean Square Deviation (RMSD) for the CMD production trajectories is shown in **Table 3S** for each sample. Samples without Cars exert higher RMSD values and suppress the excessive motion of helix-D between two conformations (L – towards the lumen, and M – towards the thylakoid membrane), termed *L-M motion* in the main manuscript.

3. RMSF and Qres values

Principal Component Analysis (PCA), or essential dynamics (ED)[44] was performed for each production trajectory of CMD runs. The single-linkage clustering method at a cut-off of 0.1nm, as embedded in GROMACS 5.0.7,[14] was used to produce an average structure for the most important cluster per sample of the CMD trajectories. For both the PCA and clustering methodologies, Ca-atom least square fit was performed prior to the application of the methods to the protein heavy atoms.

The weighted average Ca Root Mean Square Fluctuations (Ca-RMSF) per residue were calculated for the first three PCA eigenvectors of each production trajectory that was based on their respective contribution to the overall protein motion. RMSF was calculated with the average structure as reference. The Ca-RMSF values per residue give information on the flexibility of the protein under certain lumen

conditions. Complementary to RMSF, the Qres values[5,45] refer to the protein heavy atoms, they were calculated between the average structures out of the clustering method, and indicate the extend of similarity in the residual interactions (environment), at a scale from 0 to 1. Residues with high conformational similarity at different lumen buffers exert large Qres values approaching 1, whereas low conformational similarity exerts low Qres values approaching 0. The reverse is true for the (1-Qres) values. These values characterize the conformational changes in the environment of the respective residues and indicate structural differences between variant lumen ionic strengths, as presented in the main manuscript.

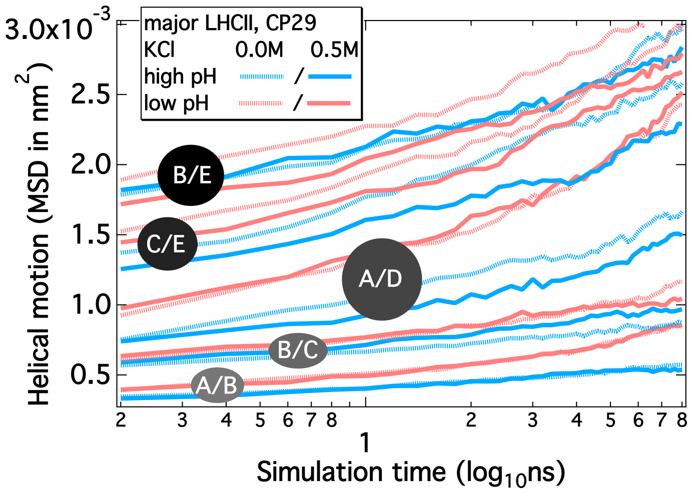

Figure 2S. The relative Mean Square Displacements (MSD) in nm^2 between pairs of helices of the wild-type (wt) major LHCII from spinach, or pea and minor CP29 from spinach, as averages over all proteins and the lumen *on the fly* perturbation production trajectories. This measures the response of the protein, upon changing the lumen buffer on the fly, from the low energization (high lumen pH) conformation to higher membrane energization states. The B/E lines are vertically shifted by $+5x10^{-4}$ and the A/D lines by $-3.5x10^{-4}$ nm^2 for clarity.

4. On the fly perturbation of the lumen buffer

A time dependent distance between the center of masses (COM) of adjacent helices in pairs can be derived throughout the resulting production trajectories. The Mean Square Displacement (MSD) is calculated on these time dependent distances and it represents a measure of the relative movement of the respective helices upon perturbing the lumen buffer. We note that the time dependent COM of the helices in each pair is calculated based on the Ca protein atoms only. The results are shown in **Figure 2S** between helices A-D, A-B, B-C, B-E and C-E upon the lumen perturbations and reveal the LHCs short time scale dynamics towards equilibria states. The results are shown as averages over all wt LHCs (with Cars) and production trajectories. While lumen exposed helix-E seems also to be exerting high mobility, the clearest separation between high and low pH lumen dynamics stands for the helix A-D motion at short time scales for all LHCs. To quantify the A-D motion, we have also probed the residues 199-207 for the major LHCII and the 216-224 residues for CP29 upon the lumen perturbation dynamics from lower to higher membrane energizations. Twenty-seven (27) atoms in each connecting loop form nine (9) triplets for the calculation of the rotational correlation functions $RCF_{rot}(t)$ over time,[46] reported in the *main manuscript*.

References

1 Liu, Z. *et al*. Crystal structure of spinach major light-harvesting complex at 2.72 Å resolution. *Nature* **428**, 287-292 (2004).

2 Standfuss, J., van Scheltinga, A. C. T., Lamborghini, M. & Kühlbrandt, W. Mechanisms of photoprotection and nonphotochemical quenching in pea light-harvesting complex at 2.5 Å resolution. *The EMBO journal* **24**, 919-928 (2005).

3 Pan, X. *et al*. Structural insights into energy regulation of light-harvesting complex CP29 from spinach. *Nature structural & molecular biology* **18**, 309-315 (2011).

4 Roberts, E., Eargle, J., Wright, D. & Luthey-Schulten, Z. MultiSeq: unifying sequence and structure data for evolutionary analysis. *BMC bioinformatics* **7**, 382 (2006).

5 Humphrey, W., Dalke, A. & Schulten, K. VMD: visual molecular dynamics. *Journal of molecular graphics* **14**, 33-38 (1996).

6 Yang, C. *et al*. The negatively charged amino acids in the lumenal loop influence the pigment binding and conformation of the major light-harvesting chlorophyll a/b complex of photosystem II. *Biochimica et Biophysica Acta (BBA)-Bioenergetics* **1777**, 1463-1470 (2008).

7 Liu, C., Rao, Y., Zhang, L. & Yang, C. Identification of the roles of individual amino acid residues of the helix E of the major antenna of photosystem II (LHCII) by alanine scanning mutagenesis. *Journal of biochemistry*, mvu028 (2014).

8 Ioannidis, N. E., Papadatos, S. & Daskalakis, V. Energizing the light harvesting antenna: Insight from CP29. *Biochimica et Biophysica Acta (BBA) - Bioenergetics* **1857**, 1643-1650, doi:http://dx.doi.org/10.1016/j.bbabio.2016.07.005 (2016).

9 Daskalakis, V., Farantos, S. C., Guallar, V. & Varotsis, C. Vibrational resonances and CuB displacement controlled by proton motion in cytochrome c oxidase. *The Journal of Physical Chemistry B* **114**, 1136-1143 (2009).

10 Wolf, M. G., Hoefling, M., Aponte-Santamaría, C., Grubmüller, H. & Groenhof, G. g_membed: Efficient insertion of a membrane protein into an equilibrated lipid bilayer with minimal perturbation. *Journal of computational chemistry* **31**, 2169-2174 (2010).

11 Mark, P. & Nilsson, L. Structure and dynamics of the TIP3P, SPC, and SPC/E water models at 298 K. *The Journal of Physical Chemistry A* **105**, 9954-9960 (2001).

12 Jorgensen, W. L., Chandrasekhar, J., Madura, J. D., Impey, R. W. & Klein, M. L. Comparison of simple potential functions for simulating liquid water. *The Journal of chemical physics* **79**, 926-935 (1983).

13 Tristram-Nagle, S., Petrache, H. I. & Nagle, J. F. Structure and interactions of fully hydrated dioleoylphosphatidylcholine bilayers. *Biophysical journal* **75**, 917-925 (1998).

14 Berendsen, H. J., van der Spoel, D. & van Drunen, R. GROMACS: A message-passing parallel molecular dynamics implementation. *Computer Physics Communications* **91**, 43-56 (1995).

15 Jorgensen, W. L., Maxwell, D. S. & Tirado-Rives, J. Development and testing of the OPLS all-atom force field on conformational energetics and properties of organic liquids. *Journal of the American Chemical Society* **118**, 11225-11236 (1996).

16 Karki, K. & Roccatano, D. Molecular Dynamics Simulation Study of Chlorophyll a in Different Organic Solvents. *Journal of Chemical Theory and Computation* **7**, 1131-1140 (2011).

17 Zoete, V., Cuendet, M. A., Grosdidier, A. & Michielin, O. SwissParam: a fast force field generation tool for small organic molecules. *Journal of computational chemistry* **32**, 2359-2368 (2011).

18 Salameh, A., Vorka, F. & Daskalakis, V. Correlation between Surface Tension and the Bulk Dynamics in Salty Atmospheric Aquatic Droplets. *The Journal of Physical Chemistry C* **120**, 11508-11518 (2016).

19 Robertson, M. J., Tirado-Rives, J. & Jorgensen, W. L. Improved peptide and protein torsional energetics with the OPLS-AA force field. *Journal of chemical theory and computation* **11**, 3499-3509 (2015).

20 Ulmschneider, J. P. & Ulmschneider, M. B. United atom lipid parameters for combination with the optimized potentials for liquid simulations all-atom force field. *Journal of Chemical Theory and Computation* **5**, 1803-1813 (2009).

21 Laganowsky, A. *et al*. Membrane proteins bind lipids selectively to modulate their structure and function. *Nature* **510**, 172-175 (2014).

22 Darden, T., York, D. & Pedersen, L. Particle mesh Ewald: An N · log (N) method for Ewald sums in large systems. *The Journal of chemical physics* **98**, 10089-10092 (1993).

23 Hess, B., Bekker, H., Berendsen, H. J. & Fraaije, J. G. LINCS: a linear constraint solver for molecular simulations. *Journal of computational chemistry* **18**, 1463-1472 (1997).

24 Miyamoto, S. & Kollman, P. A. SETTLE: an analytical version of the SHAKE and RATTLE algorithm for rigid water models. *Journal of computational chemistry* **13**, 952-962 (1992).

25 Berendsen, H. J., Postma, J. P. M., van Gunsteren, W. F., DiNola, A. & Haak, J. Molecular dynamics with coupling to an external bath. *The Journal of chemical physics* **81**, 3684-3690 (1984).

26 Bussi, G., Donadio, D. & Parrinello, M. Canonical sampling through velocity rescaling. *The Journal of chemical physics* **126**, 014101 (2007).

27 Parrinello, M. & Rahman, A. Polymorphic transitions in single crystals: A new molecular dynamics method. *Journal of Applied physics* **52**, 7182-7190 (1981).

28 Nosé, S. & Klein, M. Constant pressure molecular dynamics for molecular systems. *Molecular Physics* **50**, 1055-1076 (1983).

29 Jungwirth, P. & Winter, B. Ions at aqueous interfaces: from water surface to hydrated proteins. *Annu. Rev. Phys. Chem.* **59**, 343-366 (2008).

30 Idrissi, A., Hantal, G. & Jedlovszky, P. Properties of the liquid–vapor interface of acetone–methanol mixtures, as seen from computer simulation and ITIM surface analysis. *Physical Chemistry Chemical Physics* **17**, 8913-8926 (2015).

31 Tribello, G. A., Bonomi, M., Branduardi, D., Camilloni, C. & Bussi, G. PLUMED 2: New feathers for an old bird. *Computer Physics Communications* **185**, 604-613 (2014).

32 Jornet-Somoza, J. *et al*. Insights into colour-tuning of chlorophyll optical response in green plants. *Physical Chemistry Chemical Physics* **17**, 26599-26606 (2015).

33 Perdew, J. P., Burke, K. & Ernzerhof, M. Generalized gradient approximation made simple. *Physical review letters* **77**, 3865 (1996).

34 Zhang, Y. & Yang, W. Comment on "Generalized gradient approximation made simple". *Physical Review Letters* **80**, 890 (1998).

35 Troullier, N. & Martins, J. L. Efficient pseudopotentials for plane-wave calculations. *Physical review B* **43**, 1993 (1991).

36 Kleinman, L. & Bylander, D. Efficacious form for model pseudopotentials. *Physical Review Letters* **48**, 1425 (1982).

37 Császár, P. & Pulay, P. Geometry optimization by direct inversion in the iterative subspace. *Journal of Molecular Structure* **114**, 31-34 (1984).

38 Andreoni, W. & Curioni, A. New advances in chemistry and materials science with CPMD and parallel computing. *Parallel Computing* **26**, 819-842 (2000).

39 Elenewski, J. E. & Hackett, J. C. Cytochrome P450 compound I in the plane wave pseudopotential framework: GGA electronic and geometric structure of thiolate-ligated iron (IV)–oxo porphyrin. *Journal of computational chemistry* **34**, 1647-1660 (2013).

40 Cabral, B. J., Coutinho, K. & Canuto, S. Born-Oppenheimer molecular dynamics and electronic properties of chlorophyll-c2 in liquid methanol. *The Journal of chemical physics* **138**, 225102 (2013).

41 Liao, M.-S., Huang, M.-J. & Watts, J. D. Binding of O2 and NO to Heme in Heme-Nitric Oxide/Oxygen-Binding (H-NOX) Proteins. A Theoretical Study. *The Journal of Physical Chemistry B* **117**, 10103-10114 (2013).

42 Fiolhais, C., Nogueira, F. & Marques, M. A. *A primer in density functional theory*. Vol. 620 (Springer Science & Business Media, 2003).

43 Hirata, S. & Head-Gordon, M. Time-dependent density functional theory within the Tamm–Dancoff approximation. *Chemical Physics Letters* **314**, 291-299 (1999).

44 Mongan, J. Interactive essential dynamics. *Journal of computer-aided molecular design* **18**, 433-436 (2004).

45 Elcock, A. H. Molecular simulations of cotranslational protein folding: fragment stabilities, folding cooperativity, and trapping in the ribosome. *PLoS Comput Biol* **2**, e98 (2006).

46 Cote, Y., Senet, P., Delarue, P., Maisuradze, G. G. & Scheraga, H. A. Nonexponential decay of internal rotational correlation functions of native proteins and self-similar structural fluctuations. *Proceedings of the National Academy of Sciences* **107**, 19844-19849 (2010).
